# Altered cortical thickness and structural covariance networks in upper limb amputees: A graph theoretical analysis

**DOI:** 10.1111/cns.14226

**Published:** 2023-04-30

**Authors:** Bingbo Bao, Yi Sun, Junqing Lin, Tao Gao, Junjie Shen, Wencheng Hu, Hongyi Zhu, Tianhao Zhu, Jing Li, Zhibin Wang, Haifeng Wei, Xianyou Zheng

**Affiliations:** ^1^ Department of Orthopedic Surgery Shanghai Jiao Tong University Affiliated Sixth People's Hospital Shanghai China; ^2^ Institute of Diagnostic and Interventional Radiology Shanghai Jiao Tong University Affiliated Sixth People's Hospital Shanghai China

**Keywords:** amputation, cortical thickness, graph theoretical analysis, neuroimaging, structural covariance network

## Abstract

**Background:**

The extensive functional and structural remodeling that occurs in the brain after amputation often results in phantom limb pain (PLP). These closely related phenomena are still not fully understood.

**Methods:**

Using magnetic resonance imaging (MRI) and graph theoretical analysis (GTA), we explored how alterations in brain cortical thickness (CTh) and structural covariance networks (SCNs) in upper limb amputees (ULAs) relate to PLP. In all, 45 ULAs and 45 healthy controls (HCs) underwent structural MRI. Regional network properties, including nodal degree, betweenness centrality (BC), and node efficiency, were analyzed with GTA. Similarly, global network properties, including global efficiency (Eglob), local efficiency (Eloc), clustering coefficient (Cp), characteristic path length (Lp), and the small‐worldness index, were evaluated.

**Results:**

Compared with HCs, ULAs had reduced CThs in the postcentral and precentral gyri contralateral to the amputated limb; this decrease in CTh was negatively correlated with PLP intensity in ULAs. ULAs showed varying degrees of change in node efficiency in regional network properties compared to HCs (*p* < 0.005). There were no group differences in Eglob, Eloc, Cp, and Lp properties (all *p* > 0.05). The real‐worldness SCN of ULAs showed a small‐world topology ranging from 2% to 34%, and the area under the curve of the small‐worldness index in ULAs was significantly different compared to HCs (*p* < 0.001).

**Conclusion:**

These results suggest that the topological organization of human CNS functional networks is altered after amputation of the upper limb, providing further support for the cortical remapping theory of PLP.

## Graphical abstract

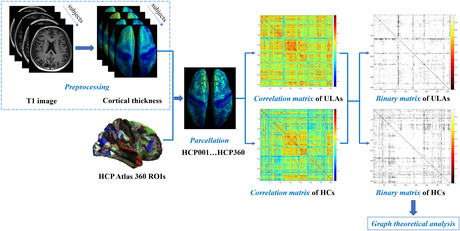

## 1 INTRODUCTION

As the prevalence of diabetes, trauma, and cancer increases, the number of amputations has correspondingly increased. In the United States, for example, there were an estimated 1.6 million amputations in 2005; this figure is expected to double to 3.6 million by 2050.^1^ Trauma is now the second most common cause of amputation.^2^ Although amputation saves many lives, the procedure is associated with serious complications, such as phantom limb pain (PLP), phantom limb sensation (PLS), residual limb pain, and neuroma.^3^ A recent study showed that the incidence of PLP after amputation can be as high as 80%.^4^ Over the past 50 years, clinicians have used pharmaceuticals, rehabilitation therapy, surgical intervention as well as 25 other methods to treat PLP after amputation, but none of these were found to reliably and effectively treat PLP.^5^ A deeper understanding of PLP is particularly important, as this knowledge will likely facilitate the search for and identification of more effective treatments for PLP.

At present, the most prominent theories about why PLP occurs and develops after amputation are the central nervous system (CNS) theory and the peripheral nervous system theory.^6^ Central to the CNS theory is the idea of cortical remapping. The cortical remapping theory (CRT) refers to the extensive functional and structural reorganizing of connectivity in the brain after limb loss.^7^, ^8^ Based on the CRT theory, some new treatment measures such as transcranial magnetic stimulation,^9^ mirror therapy,^10^ and virtual reality technology therapy^11^ have been tested and some have shown positive signs of success. Despite these recent advances, the precise CRT mechanism that fully explains why PLP arises in some patients remains unclear. Given the theoretical flux in the field, it seems reasonable to explore the possibility that a CRT mechanism might account for at least some aspects of PLP after amputation.

The rapid development of functional brain imaging over the past 30 years has fueled extensive study of the patterns of brain reorganization after amputation. CRT theory has played a major role in focusing the field, from functional to structural, from single subject studies to group studies, from study of discrete local brain regions to brain network‐level studies.^8^ Our previous imaging studies have produced new insights into brain remodeling patterns after upper limb amputation, specifically from changes in different levels of functional connectivity to changes in network connectivity.^12^, ^13^ Neural structure forms the substrate for functionality, and the integrity or change in that structure will greatly affect brain function. Currently, structural magnetic resonance imaging (MRI) studies using voxel‐based morphometry or surface‐based morphometry (SBM) have also demonstrated significant structural changes in the brain after amputation. Although these structural changes have played a prominent role in explaining postamputation reorganization,^14^, ^15^ only a few studies have been conducted using SBM to explore how cortical thickness (CTh) might change after amputation.^16^

The human brain is an extremely complex information processing network comprising 8.6*10^10^ neurons and 10*10^13^ synapses.^17^ The brain operates as an efficient network, both at the cellular level and regional level. Recently, it has become practical and revealing to analyze the brain's capacities in different neurological conditions using graph theory.^18^, ^19^ In graph theoretical analysis, mathematical network models are used to simulate neural networks and to compute the network index of the constituted network to clarify resting‐state network connectivity and changes in network functioning under various conditions.^20^

Connectivity between brain regions can be identified by measuring structural covariance networks (SCNs).^21^ A SCN of the brain can be constructed based on CTh or gray matter volume across subjects, which provide further detailed information about large‐scale brain communication.^22^, ^23^ To date, to the best of our knowledge, there are no published studies on SCNs based on CTh following unilateral amputation.

In this study, we investigated how CTh and SCNs reorganize and change in the brains of upper limb amputees (ULAs). Using graph theory analyses, we aimed to provide supplemental structural reorganization information for a richer clinical picture of the brain network characteristics of ULAs. This new information may aid in the discovery of new therapeutics and approaches for the relief of PLP of amputees and improve functional recovery using plasticity‐based therapy.

## 2 MATERIALS AND METHODS

### 2.1 Participants

This observational MRI study recruited and enrolled unilateral traumatic ULAs and age‐ and gender‐matched healthy controls (HCs) from September 2020 to December 2021. All potential participants were screened according to the following inclusion criteria: (1) aged 18–60 years; (2) unilateral upper limb traumatic amputation; and (3) time since amputation ≥6 months. Exclusion criteria were as follows: (1) non‐traumatic ULA or ULA accompanied by other limb injuries; (2) history of neurological or psychiatric disease; (3) history of using psychotropic drugs; (4) being naturally left‐handed; and (5) contraindications identified on MRI, such as incompatible heart pacemaker or a brain aneurysm, among others. The ULA group and the HC group each had 45 participants.

Handedness was assessed using the Mandarin version of the Edinburgh Handedness Inventory.^24^ Since we excluded candidate participants who were left‐handed, the dominant hand of all participants was the right. Detailed medical information was collected for all ULAs, including amputation side, amputation level (relative to elbow), time since amputation (months), PLP score, and PLS score. We used a visual analog scale based on one described by Makin et al.^14^ to assess and quantify PLP and PLS intensity (0, “no pain”; 10, “worst pain imaginable”). Of the 45 amputations, 19 were left‐side ULAs, 26 were right‐side ULAs; and of the 45, 22 were below the elbow, and 23 above the elbow. This study was approved by our hospital's ethics committee (Approval No. 2017‐034) and all participants gave informed consent prior to inclusion in this study.

### 2.2 MRI scanning and image acquisition

All images were acquired in our hospital using a 3.0‐Tesla MAGNETOM Prisma MRI scanner (Siemens, Munich, Germany) equipped with a 64‐channel phased‐array head coil. A three‐dimensional magnetization‐prepared, rapid gradient echo sequence was used to record T1‐weighted structural images using the following acquisition parameters: repetition time = 2300 ms; slice thickness = 1.0 mm; echo time = 2.46 ms; flip angle = 8°; matrix = 256 × 256 mm^2^; voxel size = 1.0 × 1.0 × 1.0 mm^3^. A total of 176 slices were acquired for each subject. The subject's head was placed in a padded foam collar to reduce head movement during scanning.

### 2.3 Data preprocessing

#### 2.3.1 Data conversion and registration

The original scanner DICOM image data were converted into NIfTI format using dcm2nii software (http://www.mricro.com) in preparation for analysis with other brain imaging tools. Prior to structural image preprocessing, we digitally flipped the brain dataset of the 19 left‐side amputees using RESTplus V1.2 (http://restfmri.net/forum/RESTplusV1.2) so that their data would be in the same orientation as those of the right‐side amputees. This would facilitate comparison. All subjects' data were processed with the Computational Anatomy Toolbox 12 (CAT12) software (http://www.neuro.unijena.de/cat/,version r1446) within SPM12 software (http://www.fil.ion.ucl.ac.uk/spm/software/spm12/, version 6225) running in a MATLAB R2013b (http://www.mathworks.com/) environment. We estimated CTh, using an automated one‐step approach based on the projection‐based thickness method,^25^ which is described below.

#### 2.3.2 Overview of data processing steps

The data processing steps mainly include brain tissue segmentation into gray matter, white matter, and cerebrospinal fluid; topological correction; spatial normalization to standard stereotactic Montreal Neurological Institute space using diffeomorphic anatomical registration using exponentiated Lie algebra; and finally performing nonlinear deformation. CTh was measured by estimating orthogonal (from surface) white matter distances and subsequently by projecting local maxima to other gray matter voxels using neighborhood relationships, which will obtain CTh mapping of every subject.^25^ Viewing quality reports obtained by CAT12, we identified participants who had a weighted average score of B's or higher at this processing stage before including their MRI data for further analysis. This is the weighted average of resolution, noise, and inhomogeneity indices. The values can range from unacceptable (*F* < 50%) to excellent (A + =100%).^26^ In all, 45 subjects from the ULA group and 45 from the HC group were analyzed further.

#### 2.3.3 CTh analysis

Connectivity between brain regions was identified by measuring SCNs.^21^ CTh using SBM analysis is a reliable and useful way to characterize brain morphometry.^25^, ^27^ Prior to making comparisons between ULAs and HCs, CTh mapping data were smoothed using a Gaussian kernel with a full‐width‐half‐maximum of 15 mm to reduce effects of affine changes. Mathematical modeling was conducted between the two groups, and a two‐sample t‐test was performed. We implemented multiple comparisons correction using a cluster‐level family‐wise error (FWE) correction protocol. A positive cluster between two groups was defined as regions of interest (ROI), and CTh was extracted from the ROI. After obtaining the ROI CTh, we conducted correlational analysis with selected clinical variables, including PLP scores, PLS scores, and elapsed time since amputation.

#### 2.3.4 SCN construction

**FIGURE 1:**
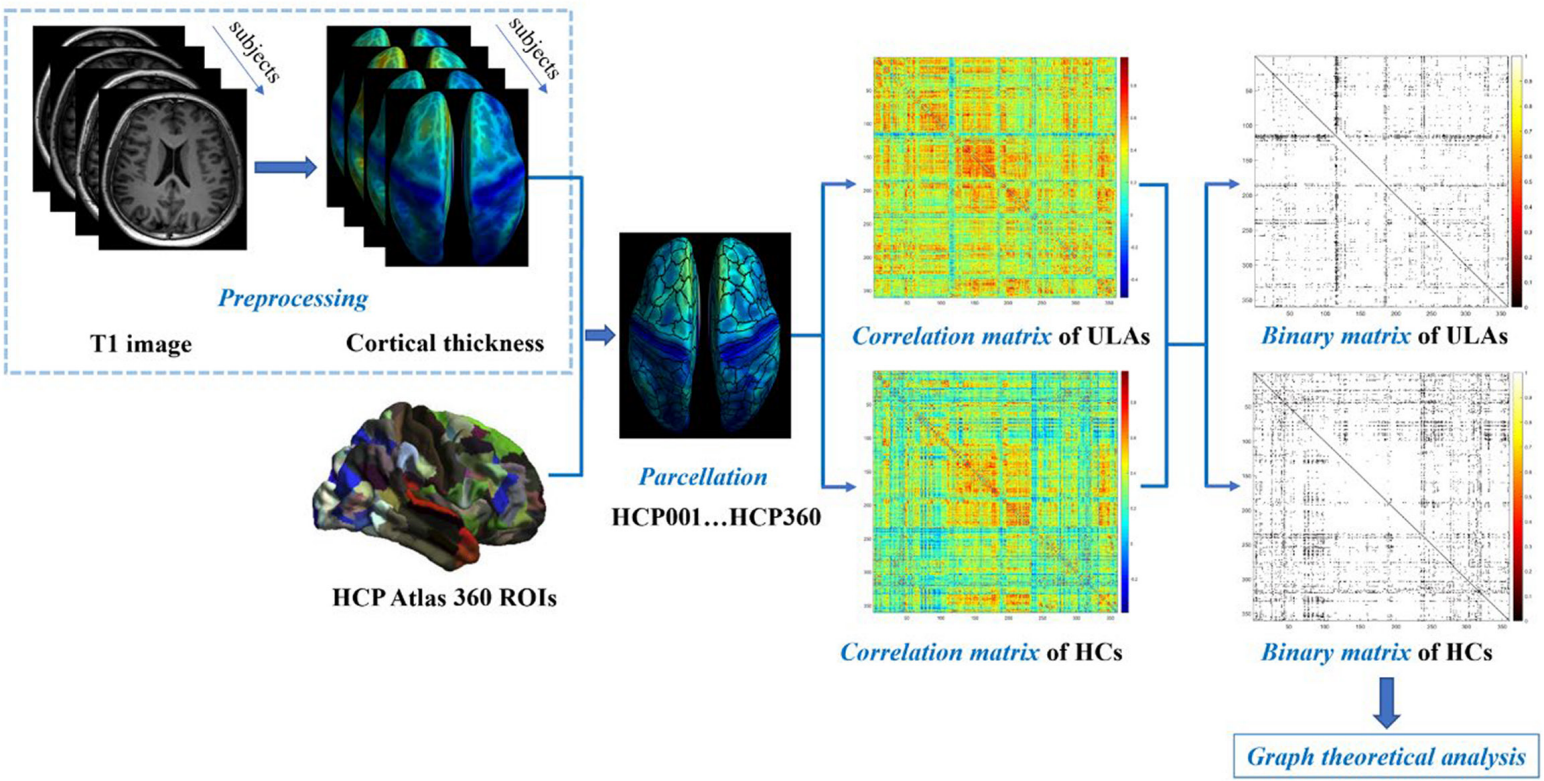
Schematic diagram of steps involved (left‐to‐right) in producing and evaluating structural covariance networks (SCNs) based on the cortical thickness (CTh) of ULA and HC participants. Green‐to‐blue areas in semi‐inflated brain surfaces (left box) represent differing CThs. The HCP 360 Atlas was used to parcellate ROIs for subsequent construction of SCNs using Pearson's correlations between each pair of corrected ROIs in ULA and HC groups. Structural correlation matrices (third from left) were calculated separately for each group to finally obtain binarized, unweighted, and undirected graph (right) used for graph theoretical analysis. Colors in structural correlation matrices represent weakest‐to‐strongest correlation (blue‐to‐red). HCP 360, Human Connectome Project Atlas; HCs, healthy controls; ROI, region of interest; ULAs, upper limb amputees.

Using the Human Connectome Project Atlas (HCP 360),^28^ we parceled the modulated CTh mapping into 360 ROIs with the CAT12 toolbox, and CTh was extracted for each ROI. SCN construction was performed with the Brain Connectivity Toolbox (http://www.brain‐connectivitytoolbox.net/)^29^ under a MATLAB environment. Pearson's correlation analysis was used to calculate the structural covariance between each pair of corrected ROIs in the ULA group and HC group. Next, we generated the structural correlation matrix, *R*. To obtain a binarized, unweighted, and undirected graph, *G*; the binarized adjacency matrix, *A*, in which *A =* [*a*_mn_], was generated. The variable *a*_mn_ was set to a value of 1 if the absolute value of *r*_mn_ between ROI_m_ and ROI_n_ was greater than a specific threshold; otherwise, *a*_mn_ was set to 0. The threshold minimum value set by the 2log(*N*)/(*N*‐1) formula (*N* = ROI numbers) and maximum value set by HC group small‐worldness index (Sigma) were >1.1.^30^ Thus, the thresholds were set over a range of network densities (Dmin = 0.02: 0.01: 0.47),^30^ because the human brain is considered as a non‐completely connected network. In graph *G*, nodes represent HCP360 ROIs, and edges represent structural connectivity between each pair of nodes. The graph theoretical analysis was applied to quantify the topological properties of *G* (Figure 1).

#### 2.3.5 Graph theoretical analysis based on SCN

Graph theoretical analysis indicators mainly include regional network analysis and global network analysis. *Regional SCN analysi*s^31^: nodal degree, nodal BC, and nodal efficiency were used to quantify the topological properties of the regional network. *Global SCN analysis* ^31^: the global efficiency (*Eglob*) of network, local efficiency (*Eloc*) of network, clustering coefficient (Cp), characteristic path length (Lp), and small‐worldness index (or *Sigma*) were used to quantify the architecture of the SCN at global network level.

### 2.4 Statistical analysis

#### 2.4.1 Baseline characteristics of participants

Baseline characteristics of participants were analyzed using GraphPad Prism 9 (GraphPad Software). Continuous variables were summarized as means ± SD, and ordinal variables as counts and percentages (%). After determining whether our data were normally distributed using the D'Agostino‐Pearson method, group differences were evaluated using two sample t‐tests for continuous variables and chi‐square tests (*χ* ^2^) for ordinal variables. *p* < 0.05 was designated as significant.

#### 2.4.2 CTh analysis

Statistical evaluations of CTh were done using two‐sample t‐tests on the right and left hemispheres separately under CAT12. Age, gender, and education were covariates for this analysis. Multiple comparisons were conducted based on the cluster‐level FWE method, with a cluster threshold of *p* = 0.001 and a corrected‐cluster significance of *p* < 0.05 (*t* > 0, mean HCs > ULAs). For positive clusters, CTh values were evaluated using two‐sample t‐tests, and *p <* 0.05 was designated as significant before determining whether CTh values were normally distributed using the D'Agostino‐Pearson method. For assessing correlations of positive clusters with clinical variables (including PLP score, PLS score, and time since amputation), we performed a two‐tailed partial correlation analysis. We used the Desikan–Killiany 40 Atlas^32^ to anatomically localize any differences in CTh between the ULA and HC groups.

#### 2.4.3 Graph theoretical analysis

Graph theoretical analysis for group differences of topological properties in SCNs was based mainly on permutation test theory. For evaluation of the real‐world SCNs of ULAs and HCs, topological properties under the real‐world SCNs were calculated with different densities. Subsequently, we reassigned the two groups randomly to obtain a new permutation group (subject numbers consistent with the original group), and then calculated the topological properties in the permutation group. In all, 5000 random permutations were repeated, producing 5000 random SCNs and their corresponding topological properties. In addition, due to different sparsity, we statistically compared the areas under the curve (AUC) of the topological properties under different sparsity values. Then the AUC differences of the topological properties between the ULAs and HCs in the real‐world SCNs were compared with the 5000 random SCNs. Differences greater than the 95% confidence intervals between the two groups were considered as significantly different. The significance level was set at *p <* 0.05 after false discovery rate correction for multiple comparisons of 360 ROIs based on the HCP 360 Atlas in the regional network analysis. Similarly, *p <* 0.05 was used for analyses of the global network.

## 3 RESULTS

### 3.1 Clinical and demographic characteristics of participants

Table 1 summarizes the basic characteristics of the ULA and HC groups. ULA and HC participants' clinical and demographic characteristics were statistically indistinguishable (all *p >* 0.05). Amputations were almost evenly distributed above and below the elbow, with more patients having right‐side amputations. On average, all testing and imaging took place about 2 years after amputation, a time when the mean PLS score was above 6 on a scale of 1–10 (10 being the worst imaginable pain), and the mean PLP score was just under 4 (Table 1). All the structural images obtained during the approximately 1‐year enrollment period had good quality scores (at least B+), as assessed by CAT12 quality reports.

**TABLE 1:** Demographic and clinical characteristics of participants.

Characteristic	ULA (*n* = 45)	HC (*n* = 45)	*χ* ^2^ or *t*	*p*
Age	44.47 ± 8.86^a^	45.16 ± 9.30	0.360	0.720
Education (year)	7.51 ± 4.33^a^	7.89 ± 4.14	0.423	0.673
Female/Male	11/34	12/33	0.242	0.809
Time since amputation (mo)	25.31 ± 16.70^a^			
Amputation side (left/right)	19/26			
Amputation location (above/below elbow)	23/22			
PLS intensity^b^	6.73 ± 2.90^a^			
PLP intensity^b^	3.93 ± 2.72^a^			

Abbreviations: HCs, healthy control group; PLP, phantom limb pain; PLS, phantom limb sensation; ULA, upper limb amputee group.

^a^ SD.

^b^ Intensity was self‐rated on a linear scale, with 0 representing “no pain” and 10 representing the “worst pain imaginable”.

### 3.2 Group differences in CTh

**FIGURE 2:**
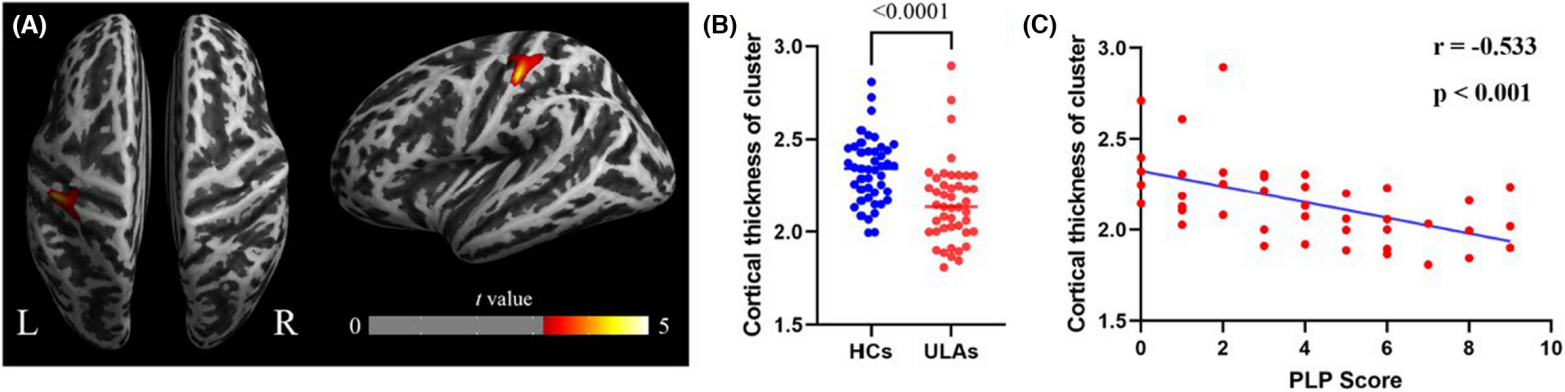
Localized decrease in cortical thickness (CTh) after upper limb amputation. (A) Surface of the left‐side of the semi‐inflated brain (right) of a representative ULA patient showing location of decrease, predominately in postcentral gyrus. Color scale indicates greater t‐values toward yellow color. Cluster of CTh decreased significantly after amputation (95% left postcentral and 5% left precentral) (Desikan–Killiany 40 Atlas) (*p* < 0.05; cluster‐level FWE corrected). (B) Quantitation of significant CTh clusters. Individual subjects' (*n* = 45) CTh data (dots) overlaid on group means (colored horizonal lines). (C) Quantitative association between clinical PLP score and CTh of ULA subjects (*n* = 45). FEW, family‐wise error; L, Left; PLP, phantom limb pain; R, Right; ULA, upper limb amputee.

We found a significant decrease in CTh in the ULA group in two places in the contralateral hemisphere (relative to the amputation side). Compared to the HC group, the CTh of the ULA group was significantly reduced in the left postcentral and precentral gyri (*p* < 0.05; FWE corrected; Figure 2A,B; Table 2). Figure 2A shows the location of the cluster of CTh changes plotted on a cortical surface image. According to the Desikan–Killiany 40 Atlas,^32^ most of the change in CTh was localized in the postcentral gyrus. Figure 2B and Table 2 present quantitative information about the positive clusters (i.e., clusters above the group mean). Although we observed CThs of some ULA patients overlapped with the CThs of HC participants (Figure 2B), the CTh of the ULA group was significantly less than that of the HC group (*p* < 0.001).

**TABLE 2:** Cortical locations of significant differences in CTh between ULA and HC.

Overlap of atlas region^a^	Hemisphere	Cluster size	*p* Value (FWE corrected)	T
95% postcentral gyrus	Left	1393	0.00001^b^	4.6
5% precentral gyrus	Left			

^a^ Desikan–Killiany 40 Atlas (Desikan et al., 2006) was used to anatomically localize any differences in CTh.

^b^ FWE corrected at cluster level; HCs > ULAs.

Abbreviations: CTh, cortical thickness; FWE, family‐wise error; HCs, healthy controls; ULAs, upper limb amputees.

Correlational analysis between clinical manifestations of ULA and CTh showed that there was a significant negative correlation between the CTh‐positive clusters and the PLP score (*r* = −0.533, *p* < 0.001; Figure 2C). Thus, with decreasing CTh, phantom pain was greater in ULAs.

### 3.3 Group differences in regional SCN analysis

**FIGURE 3:**
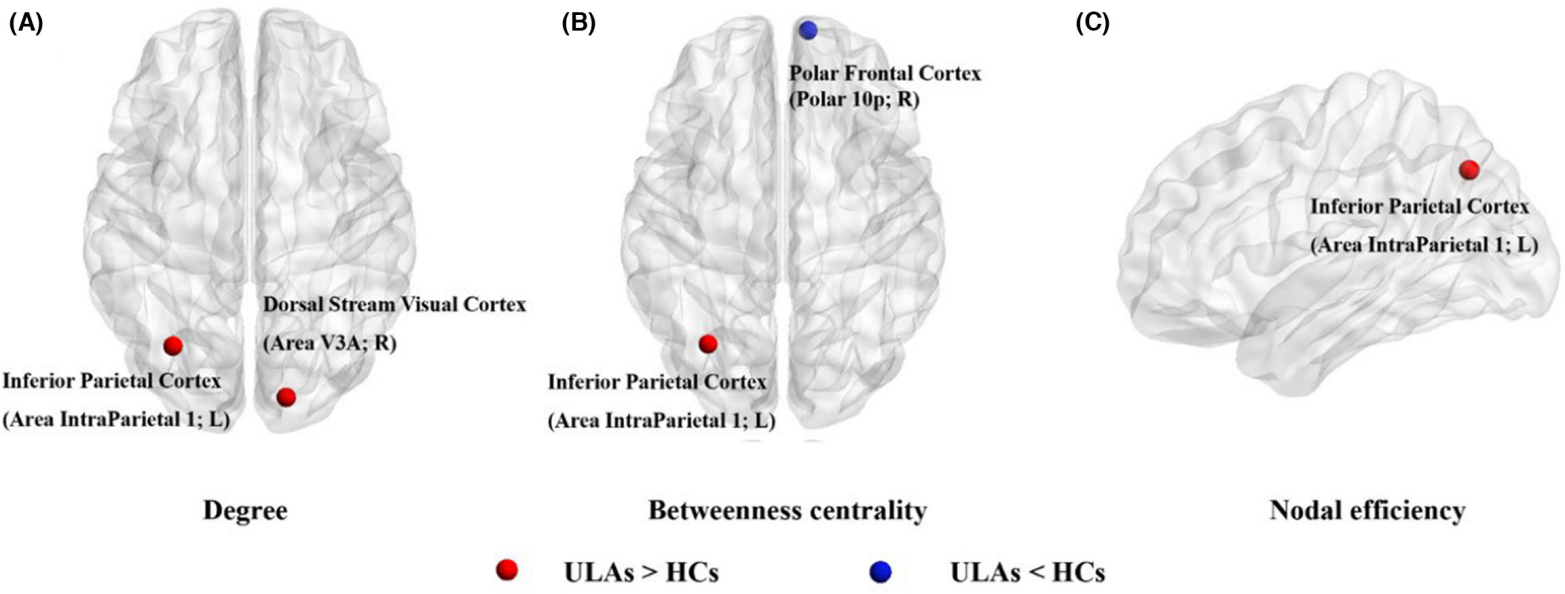
Changes in SCN regional topological properties of neocortex after amputation. (A) ULA group had a higher nodal degree in dorsal stream visual cortex (area V3A) and inferior parietal cortex (area intraparietal 1) compared with HC group (*p* < 0.005, uncorrected); representation of brain regions is based on the HCP360 Atlas. (B) ULA group had larger *BC* values in inferior parietal cortex (area intraparietal 1) and lower *BC* values in orbital and polar frontal cortex (polar 10p) compared with HC group (*p* < 0.005, uncorrected). (C) ULA group had greater nodal efficiency in inferior parietal cortex (area intraparietal 1) compared with HC group (*p* < 0.005, uncorrected). BC, betweenness centrality; L, Left; R, Right; HCs, healthy controls; SCN, structural covariance network; ULA, upper limb amputee.

We also found group differences in SCNs in the regional SCN topological analysis.^31^ Table 3 summarizes information about the group differences in the regional SCN analysis. Compared with the HC group, in the ULA group the nodal degrees increased in the right dorsal stream of visual cortex (Area V3A; *p* = 0.003, uncorrected) and in the left inferior parietal cortex (area intraparietal 1; *p* < 0.001, uncorrected) (Figure 3A). Also, the *BC* of the left inferior parietal cortex (area intraparietal 1; *p* = 0.001, uncorrected) increased, whereas the *BC* of the right orbital and polar frontal cortex (Polar 10p; *p* = 0.004, uncorrected) decreased (Figure 3B). The nodal degrees in the left inferior parietal cortex (area intraparietal 1; *p* = 0.001, uncorrected) also increased in ULAs (Figure 3C).

**TABLE 3:** Group differences in regional SCN analysis.

		*p* Values (uncorrected)		
Contrast	Side	Degree	Betweenness centrality	Nodal efficiency
ULAs > HCs				
Dorsal stream visual cortex (Area V3A)	R	0.003	–	–
Inferior parietal cortex (area intraparietal 1)	L	<0.001	0.001	0.001
ULAs < HCs				
Orbital and polar frontal cortex (Polar 10p)	R	–	0.004	–

Abbreviations: HCs, healthy controls; SCN, structural covariance network; ULAs, upper limb amputees.

### 3.4 Group differences in global SCN analysis

**FIGURE 4:**
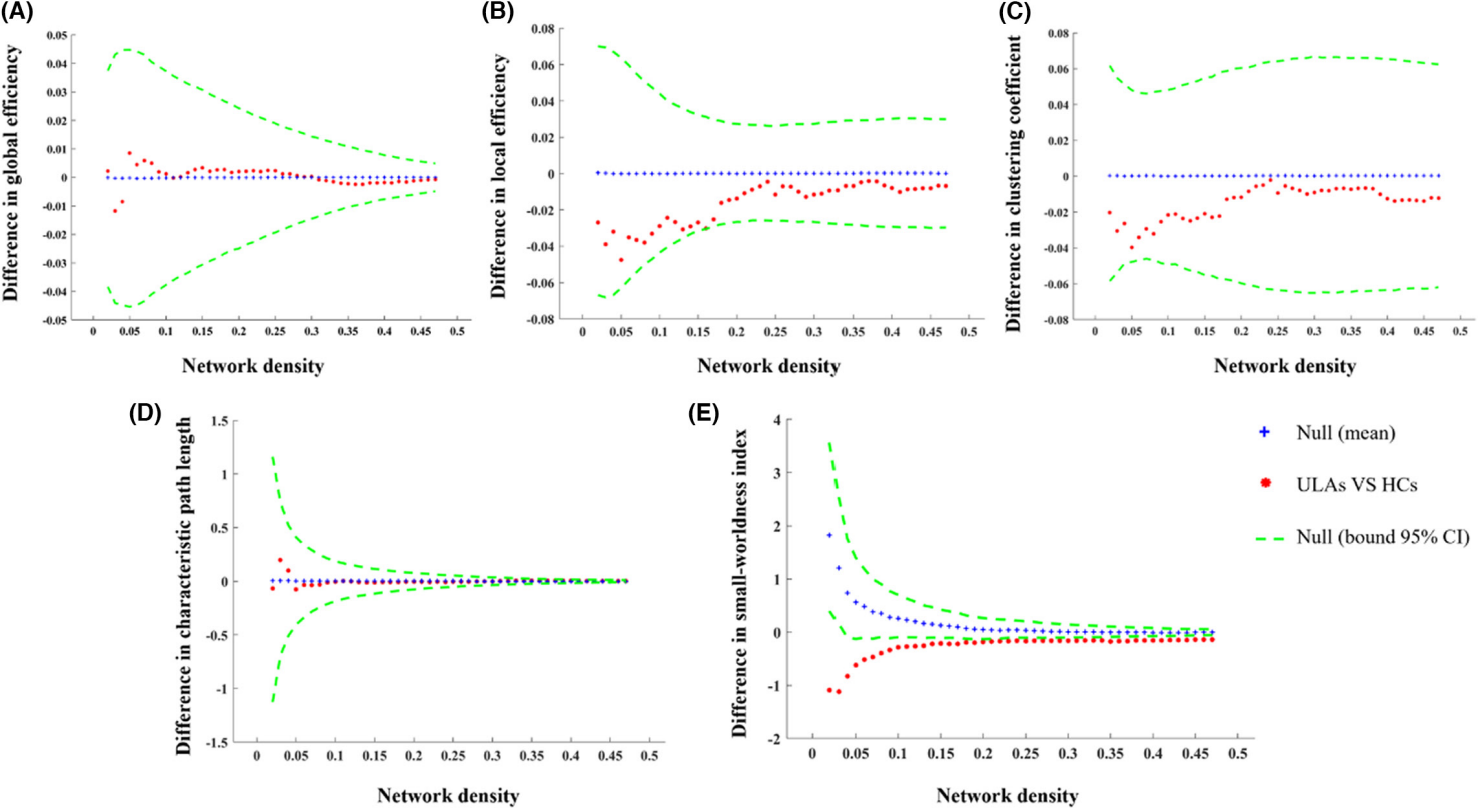
Differences between ULAs and HCs in global network properties at different densities. (A) Global efficiency. (B) Local efficiency. (C) Clustering coefficient. (D) Characteristic path length. (E) Small‐worldness index. There were no intergroup differences in global efficiency (A), local efficiency (B), clustering coefficient (C), and characteristic path length (D) (all *p* > 0.05). However, ULAs showed lower small‐worldness index values at all network density ranges compared to HCs (*p* < 0.001). The blue pluses (+) indicate intergroup differences in the permutation random structural covariance network (SCN), whereas green dash lines denote 95% confidence intervals (CIs). The red asterisks (*) indicate intergroup differences of real SCNs. Red asterisks falling outside of the 95% CIs indicate that the density at which the intergroup difference of real SCN was significant at *p* < 0.05, which means that there is a significant difference between ULA and HC groups. The positive values indicate ULAs > HCs, and negative values indicate ULAs < HCs. HCs, healthy controls; ULAs, upper limb amputees.

**FIGURE 5:**
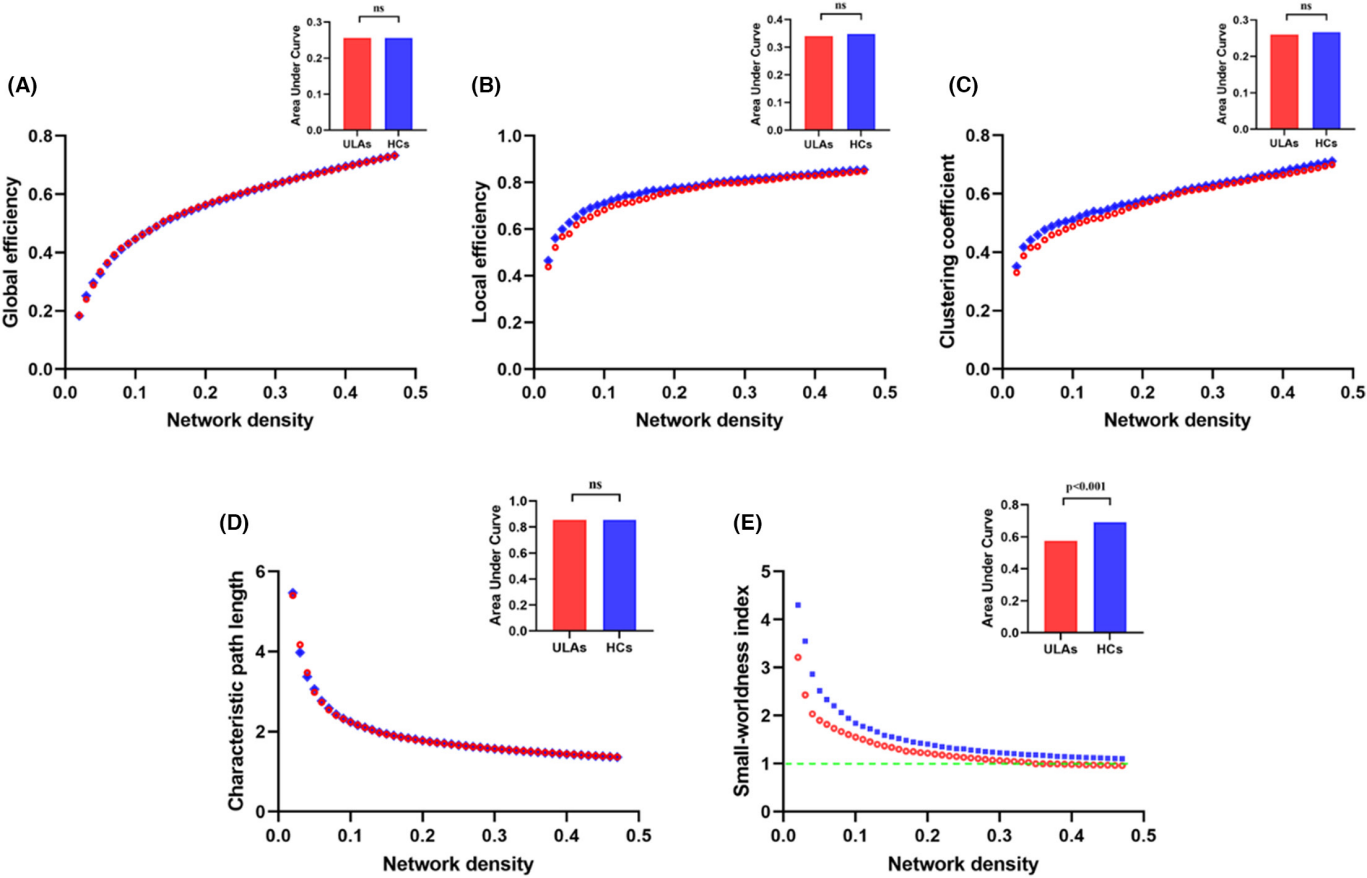
Changes in the global real‐world structural covariance network properties of ULAs and HCs at different densities. (A) Global efficiency. (B) Local efficiency. (C) Clustering coefficient. (D) Characteristic path length. (E) Small‐worldness index. The real‐world structural covariance network of ULAs showed a small‐world property (Sigma >1) only at sparsity values ranging from 2% to 34%. The small‐worldness index AUC at different network densities in the real network was statistically different between the ULAs and the HCs (*p* < 0.001). AUC, area under the curve; HCs, healthy controls; ULAs, upper limb amputees.

There were no group differences in *Eglob*, *Eloc*, *Cp*, *and Lp* of the global network analysis (all *p* > 0.05; Table 4; Figure 4A–D). Changes in *Eglob*, *Eloc*, Cp, *and* Lp over a range of network densities in real‐world SCNs are shown in Figure 5A,E. For ULAs, real‐world SCNs had a small‐world topology over densities ranging from 2% to 34% (small‐worldness index/Sigma >1; Figure 5E). Compared with HCs, ULAs had a smaller small‐worldness index under the AUC (*p* < 0.001; Table 4; Figures 4E and 5E).

**TABLE 4:** Intergroup differences in global SCN analysis.

Global network properties	*p* Values
Global efficiency	0.141
Local efficiency	0.141
Clustering coefficient	0.536
Characteristic path length	0.996
Small‐worldness index	<0.001

Abbreviation: SCN, structural covariance network.

## 4 DISCUSSION

The intrinsic brain remodeling that occurs after amputation in patients has been extensively studied and validated in recent years. Since PLP, PLS, and neuromas often emerge after amputation,^3^ a major focus of this research has been to elucidate pathophysiological mechanisms of pain after amputation and how this relates to functional CNS remodeling. The CNS theory of PLP emergence after amputation has stimulated new therapeutic approaches, such as virtual reality therapy and mirror therapy, which has provided some support for the CNS theory and some relief of PLP for patients. However, a CNS mechanism as a basis for PLP is still incompletely understood and imprecisely formulated. Here, we used MRI and graph theoretical analysis to show that particular parameters of the topological organization of human CNS functional networks are altered after amputation of the upper limb, further specifying a possible CNS mechanism underlying PLP.

In recent years, structural MRI has been widely used to better understand mechanisms linking structure and function. This has been a profitable approach to unraveling how changes in brain structure of amputees are linked to changes in function and sensation. In a study of 18 ULAs, for example, Makin et al. found that gray matter volume decreased in the sensorimotor cortex after amputation, but in patients with less gray matter volume loss, PLP intensity was less.^14^ Another study of 21 ULAs showed a significant decrease in gray matter volume in the motor cortex representing the amputated limb and an increase in the dorsal and ventral visual cortex.^15^ Jiang et al. also found that the CTh of the V5/middle temporal (V5/MT+) visual area was significantly reduced after amputation in 48 unilateral lower limb amputees, and CTh was negatively correlated with the time since amputation.^16^ Together, these structural MRI studies demonstrated that structural change occurs in the brain after amputation.

Similarly, in this study, we found specific structural changes in the contralateral hemisphere of ULAs 2 years after traumatic amputation. CTh decreased in the expected brain areas after upper limb amputation, and this reduction correspondingly changed regional functional networks. We found a significant brain cluster in ULAs localized to 95% postcentral gyrus and 5% precentral gyrus, based on the Desikan–Killiany 40 Atlas.^32^ The postcentral gyrus contains the primary somatosensory cortex responsible for proprioception, which processes various somatic sensations from the body, including touch, pain, temperature, and pressure.^33^, ^34^ The precentral gyrus contains the primary motor cortex responsible for the control of voluntary motor movement.^35^ Based on present functional understanding of the postcentral and precentral gyri, it is not surprising that CTh decreased in the contralateral brain region after upper limb amputation following deprivation of limb sensory input and lack of corresponding limb movement.

In our previous studies, both functional connectivity and functional brain network analyses demonstrated that significant changes also occur in this region.^12^, ^13^ These findings from this study provide further evidence supporting the coupling of function and structure, which will guide us in future studies of the CRT after amputation. Interestingly, our correlation analysis with clinical manifestations showed that the decrease in CTh was negatively correlated with PLP score, which suggests that lower CTh was associated with more intense PLP.

One hypothesis to account for this structural and sensational change is that the preservation of CTh in postcentral and precentral gyrus after amputation in ULAs mitigates to some degree the occurrence or intensity of PLP. This notion is consistent with the finding that repeated transcranial magnetic stimulation after traumatic amputation inhibits CTh loss in a top‐down mechanism, and it provides significant analgesia in PLP patients.^36^ In a bottom‐up mechanism, targeted muscle reinnervation technology combined with an intelligent prosthesis produces continuous sensory nerve input and motor function to preserve CTh after amputation and thus alleviates PLP.^37^, ^38^ Both approaches are ripe for further research.

From the brain network perspective, after amputation the sensorimotor network undergoes structural and functional reorganization,^39^ as do the default mode network^40^ and the dorsal attention network.^13^ Because the brain is an extremely complex and highly efficient network, there must be functional coupling or antagonistic effects in functional areas.^41^ This implies that network‐sensitive neuroimaging methods could reveal changes in functional connectivity networks after sensorimotor perturbations or CNS neurodegenerative disease.^21^ Using graph theoretical analysis gives us the opportunity to use structural data such as CTh to analyze various network changes from different perspectives.^16^ CTh‐based structural covariant networks will exhibit topological changes in the brain network after upper limb amputation from a different perspective and will provide a new perspective toward brain plasticity after amputation.

To our knowledge, our study is the first to conduct, on brain network level, a graph theoretic analysis of SCNs in ULAs based on CTh. Our SCN results showed that compared with HCs, ULAs showed increased nodal degree, *BC*, and nodal local efficiency of intraparietal 1 area following contralateral limb amputation. These regional properties revealed in inferior parietal cortex of ULAs all reflect the importance of this node to the network. In human intraparietal sulcus,^42^ numerous studies have shown that this region mediates the precise coordination of finger sensorimotor integration.^43^, ^44^, ^45^ Interestingly, almost all ULAs experience PLS, with the lost limb and/or finger being curled and uncorrectable.^46^ Whether this painful PLP experience is directly related to the abnormally enhanced role of this region in the network remains to be demonstrated.

In the global SCN analysis, ULAs showed weaker small‐world network properties, a parameter that reflects the brain's ability to transmit and process information efficiently. Recent research has shown that amputees are increasingly reluctant to engage in social and other activities.^47^, ^48^ Further research is needed to determine whether changes in the network of small‐world properties are related to amputees' reduced desire to engage in social and other activities after amputation. In conclusion, our findings provide a new perspective on brain network remodeling after amputation and suggest many new directions for research.

Although our study of CTh in ULAs provided some new findings and insights about CNS network changes after amputation, it has some limitations. First, our sample size was rather small. Second, additional factors we did not study, such as intact hand use, use of a prosthesis, and amputation of dominant limb versus nondominant limb, may have additionally affected brain reorganization. Given these limitations, further studies of ULAs need to be conducted with larger numbers of subjects.

## 5 CONCLUSION

In summary, this study of CTh and SCNs in amputees suggests that the topological organization of human brain functional networks is altered after amputation, which provides more support for the CRT of the phantom sensations experienced by amputees. Moreover, these new insights into understanding the functional substrates of brain network remodeling after amputation of the upper limb suggest that brain network analysis via graph theoretical analysis could be useful for understanding other kinds of cortical deafferentations.

## AUTHOR CONTRIBUTIONS

Xianyou Zheng and Haifeng Wei was responsible for study design and manuscript revision. Bingbo Bao and Yi Sun were responsible for data collection and analysis. Bingbo Bao was responsible for manuscript writing. Bingbo Bao and Yi Sun contributed equally to this manuscript. All the authors critically reviewed the content of the manuscript. All authors read and approved the final manuscript.

## FUNDING INFORMATION

This study was supported by Shanghai Municipal Education Commission‐Gaofeng Clinical Medicine Grant (grant number 20161429), National Natural Science Foundation of China (grant number 81974331, 82172421).

## CONFLICT OF INTEREST STATEMENT

The authors declare that they have no conflicts of interests.

## Data Availability

The raw data supporting the conclusions of this article will be made available by the authors with reasonable request.
